# Metabolic Pathways Involved in Carbon Dioxide Enhanced Heat Tolerance in Bermudagrass

**DOI:** 10.3389/fpls.2017.01506

**Published:** 2017-09-19

**Authors:** Jingjin Yu, Ran Li, Ningli Fan, Zhimin Yang, Bingru Huang

**Affiliations:** ^1^College of Agro-grassland Science, Nanjing Agricultural University Nanjing, China; ^2^Department of Plant Biology and Pathology, Rutgers, The State University of New Jersey, New Brunswick NJ, United States

**Keywords:** bermudagrass, elevated CO_2_, heat stress, metabolites, protein

## Abstract

Global climate changes involve elevated temperature and CO_2_ concentration, imposing significant impact on plant growth of various plant species. Elevated temperature exacerbates heat damages, but elevated CO_2_ has positive effects on promoting plant growth and heat tolerance. The objective of this study was to identify metabolic pathways affected by elevated CO_2_ conferring the improvement of heat tolerance in a C_4_ perennial grass species, bermudagrass (*Cynodon dactylon* Pers.). Plants were planted under either ambient CO_2_ concentration (400 μmol⋅mol^-1^) or elevated CO_2_ concentration (800 μmol⋅mol^-1^) and subjected to ambient temperature (30/25°C, day/night) or heat stress (45/40°C, day/night). Elevated CO_2_ concentration suppressed heat-induced damages and improved heat tolerance in bermudagrass. The enhanced heat tolerance under elevated CO_2_ was attributed to some important metabolic pathways during which proteins and metabolites were up-regulated, including light reaction (ATP synthase subunit and photosystem I reaction center subunit) and carbon fixation [(glyceraldehyde-3-phosphate dehydrogenase, GAPDH), fructose-bisphosphate aldolase, phosphoglycerate kinase, sedoheptulose-1,7-bisphosphatase and sugars) of photosynthesis, glycolysis (GAPDH, glucose, fructose, and galactose) and TCA cycle (pyruvic acid, malic acid and malate dehydrogenase) of respiration, amino acid metabolism (aspartic acid, methionine, threonine, isoleucine, lysine, valine, alanine, and isoleucine) as well as the GABA shunt (GABA, glutamic acid, alanine, proline and 5-oxoproline). The up-regulation of those metabolic processes by elevated CO_2_ could at least partially contribute to the improvement of heat tolerance in perennial grass species.

## Introduction

Global climate changes involve elevated temperature and CO_2_ concentration, imposing significant impact on plant growth (Kirkham, 2011). During this century, global temperatures are predicted to rise by 2–5°C; atmospheric CO_2_ concentration has increased by 100 μmol mol^-1^ since the beginning of the industrialized era and the concentration is predicted to continue rising at a rate of approximately 2 μmol mol^-1^ per year (Intergovernmental Panel on Climate Change [IPCC], 2007). Previous research has shown that elevated CO_2_ promotes plant growth under optimal growing temperatures in various plant species (Hamerlynck et al., 2000; Prasad et al., 2002; Qaderi et al., 2006). Recent research also found that elevated CO_2_ has positive effects on promoting heat tolerance in terms of vegetative growth in C_3_ species, such as rice (*Oryza sativa*) (Sujatha et al., 2008; Figueiredo et al., 2015; Lai et al., 2015), wheat (*Triticum aestivum*) (Bencze et al., 2005; Alonso et al., 2009), and cool-season perennial grass species (Yu et al., 2012a, 2014) and C_4_ plant species, such as *Bouteloua gracilis* (Read and Morgan, 1996), peanut (*Arachis hypogaea*) (Prasad et al., 2010), grain sorghum (*Sorghum bicolor*) (Prasad et al., 2006) and maize (*Zea mays*) (Abebe et al., 2016). The mechanisms regulating elevated CO_2_ effects on C_3_ plant species have been reported, which have been associated with enhanced cellular expansion and cell division resulted from increased carbohydrate availability and changes in proteins and gene transcript levels (Pritchard et al., 1999; Kirkham, 2011; Morgan et al., 2011; Huang and Xu, 2015). However, metabolic factors underlying elevated CO_2_ improvement of heat tolerance in C_4_ perennial grass species are not well understood.

Metabolic and proteomic analysis mostly in C_3_ plant species demonstrated that elevated CO_2_ causes changes in various metabolic processes or pathways such as photosynthetic carbon fixation, respiratory metabolism, cellular growth, and stress defense (Fukayama et al., 2009; Yu et al., 2012a, 2014; Burgess and Huang, 2014, 2016). The improved heat tolerance by doubling ambient CO_2_ concentration in C_3_ grass species, such as tall fescue (*Festuca arundinacea*), has been attributed to increases in the accumulation of metabolites, such as organic acids (shikimic acid, malonic acid, glyceric acid, threonic acid, galactaric acid, and citric acid), sugars (sucrose and maltose) and amino acids (valine, serine, and 5-oxoproline) involved in photosynthesis, respiration and amino acid metabolism (Yu et al., 2012a). In addition, doubling ambient CO_2_ concentration significantly increased the accumulation of soluble leaf carbohydrates and activity of adenosine-5′-diphosphoglucose pyrophosphorylase under high temperature in kidney bean (*Phaseolus vulgaris*) (Prasad et al., 2004). Proteomic profiling of tall fescue exposed to elevated CO_2_ concentration under heat stress found increased abundance of proteins associated with functions of photosynthetic light reaction, electron transport carrier molecule, ATP generation enzyme and antioxidant system (Yu et al., 2014). It has been reported that C_4_ plant species are generally less responsive to elevated CO_2_ than C_3_ species when they are exposed to their respective optimal temperature conditions (Kirkham, 2011; Huang and Xu, 2015). Mechanisms of elevated CO_2_-induced stimulation of photosynthesis in C_3_ plants were mainly associated with changes in electron transport during in light reaction as well as capacity for carbon fixation and assimilation during dark respiration (Yu et al., 2012b, 2014; Huang and Xu, 2015). However, the key changes in metabolites and proteins induced by elevated CO_2_ in C_4_ plants under heat stress have not yet to be determined.

The objective of the current study was to identify metabolic pathways affected by elevated CO_2_ conferring the improvement of heat tolerance in a C_4_ perennial grass species, bermudagrass (*Cynodon dactylon*) widely used as forage and turfgrass species. Understanding changes of metabolites and proteins in C_4_ species in response to elevated CO_2_ concentration will provide new insights to mechanisms about elevated CO_2_-mitigated effects on heat stress.

## Materials and Methods

### Plant Materials and Growth Conditions

Stolons of bermudagrass (cv. ‘Tifway’) plants were collected from the research farm at Nanjing Agricultural University in Nanjing, China, and transplanted into pots (20 cm in diameter and 20 cm in depth) filled with a mixture of soil and sand (soil: sand = 1:1, v/v). Plants were grown in a greenhouse with average temperature of 30/22°C (day/night), natural sunlight and irrigated once a week with half-strength Hoagland’s nutrient solution (Hoagland and Arnon, 1950) to establish canopy and roots for 2 months. During this period, plants were trimmed once a week to keep a canopy height of 4–5 cm. After establishment, plants were transferred to growth chambers (Xubang, Jinan, Shandong province, China) with the temperature of 30/25°C (day/night), 70% relative humidity, photosynthetically active radiation of 650 μmol⋅m^-2^⋅s^-1^ and a 12-h photoperiod.

### Experimental Design and Treatments

The CO_2_ concentrations set-up and control in growth chambers followed the same designed as described in Yu et al. (2012a,b). Each CO_2_ treatment was imposed in four growth chambers on September 1, 2015. In order to evaluate the long-term effects of elevated CO_2_, plants were grown under the two CO_2_ concentrations for 70 days prior to the exposure to heat stress. Plants grown under either CO_2_ treatment was then exposed to (45/40°C) (heat stress) or 30/25°C (non-stress control) in two growth chambers on November 9, 2015 until December 8, 2015. Plants were randomly relocated in each chamber twice per week to avoid confounding effects of environmental variation between different chambers. The CO_2_ concentration inside each growth chamber was controlled by an automated, open-chamber CO_2_ control system connected to a gas tank containing 100% CO_2_ (Yu et al., 2015).

The experiment was arranged as factorial design with two CO_2_ concentrations (ambient CO_2_ concentration at 400 ± 10 μmol⋅mol^-1^ and elevated CO_2_ concentration at 800 ± 10 μmol⋅mol^-1^) and two temperature treatments [30/25°C (day/night, optimal temperature control) and 45/40°C (day/night, heat stress)]. Each treatment was repeated in four pots of plants (four replicates).

### Measurements of Physiological Indexes

Leaf net photosynthetic rate (*P*_n_) was determined by inserting 4–5 individual leaves (second full-expanded from the top) collected from each pot to a 6 cm^2^ cuvette with a portable infrared gas analyzer (Li-6400, LI-COR, Inc., Lincoln, NB, United States). Leaves were placed in a leaf chamber with a built-in red and blue light source of the Li-6400 with the light intensity of 800 μmol photon⋅m^-2^⋅s^-1^.

For leaf chlorophyll content (Chl), 0.2 g of fresh leaves were detached from plants and then immersed in dimethyl sulfoxide (DMSO) in dark for at least 72 h for a complete extraction of total chlorophyll. The absorbance of the Chl extract was measured at wavelengths of 663 and 645 nm, respectively, by a spectrophotometer (Ultrospec 2100 pro, Biochrom Ltd., Cambridge, England) to calculate Chla and Chlb content. Chl was determined as described by Arnon (1949). For photochemical efficiency (*F*_v_/*F*_m_), chlorophyll fluorescence (the ratio of variable to maximum fluorescence as *F*_v_/*F*_m_) was measured by a fluorescence induction monitor (Bioscientific Ltd., Herts, United Kingdom) following 30 min dark acclimation through leaf tips.

### Metabolites Extraction and Quantification

The extraction procedure was conducted following the method of Roessner et al. (2000) and Rizhsky et al. (2004). Leaf samples collected at 28 days of treatment were collected and immediately frozen in liquid nitrogen, then stored at -80°C for metabolic profiling analysis. For each sample, frozen dry leaves were ground to a fine powder with liquid nitrogen, and then 25 mg of powder was transferred into a 10 mL microcentrifuge tubes, and extracted in 1.4 mL of 80% (v/v) aqueous methanol at 23°C for 2 h. Ribitol solution of 10 μL (2 mg⋅mL^-1^ water) as an internal standard was added prior to incubation. Then, extraction was performed in a water bath at 70°C for 15 min. Tubes were centrifuged for 30 min at 9660 *g*n and the supernatant was decanted into new tubes, 1.4 mL of water and 0.75 mL of chloroform were added. The mixture was vortexed thoroughly and centrifuged for 15 min at 5025 *g*n and then 1 mL of the polar phase (methanol/water) was pipetted into HPLC vials and dried in a centrifugal concentrator (Centrivap, Labconco Corporation, Kansas City, MO, United States). The dried polar phase was methoximated with 80 μL of 20 mg⋅mL^-1^ methoxyamine hydrochloride at 30°C for 90 min and then was trimethylsilylated with 80 μL N-methyl-N-(trimethylsilyl) trifluoroacetamide (MSTFA) (with 1% TMCS) for 60 min at 70°C.

The Gas Chromatography-Mass Spectrometer (GC-MS) analysis was modified from Qiu et al. (2007). The derivatized extracts were analyzed with a GC coupled with a TurboMass-Autosystem XL MS (Perkin Elmer Inc., Waltham, MA, United States). A 1 μL extracts was injected into a DB-5MS capillary column (30 m × 0.25 mm × 0.25 μm, Agilent J & W Scientific, Folsom, CA, United States). The inlet temperature was held at 260°C. After a 6.5 min solvent delay, initial GC oven temperature was maintained at 60°C; 1 min after injection, the GC oven temperature was raised to 280°C at a rate of 5°C⋅in^-1^, and finally maintained at 280°C for 15 min. The injection temperature was set at 280°C and the ion source temperature was adjusted to 200°C. Helium was used as the carrier gas with a constant flow rate of 1 mL⋅min^-1^. The measurements were performed through electron impact ionization (70 eV) in the full scan mode (m/z 30–550). The detected metabolites were identified with Turbomass 4.1.1 software (PerkinElmer Inc., Waltham, MA, United States). For GC/MS results, compounds were identified based on retention time (RT) and comparison with reference spectra in mass spectral libraries.

### Protein Extraction and Quantification

Leaf samples were collected from each tube at 28 days, immediately frozen in liquid nitrogen, then ground into fine powder and stored at -80°C until analysis. Proteins were extracted using the trichloroacetic acid (TCA)/Acetone method described from Xu and Huang (2008). Leaf powder samples (0.5 g) were homogenized on ice in precipitation solution (10% TCA and 0.07% 2-mercaptoethanol in acetone) for 10 min and then incubated at -20°C for 2 h. The protein pellet was collected and washed with cold acetone containing 0.07% 2-mercaptoethanol until the supernatant was colorless. The pellet was then vacuum-dried and suspended in resolubilization solution [8 M urea, 2 M thiourea, 2% CHAPS, 1% dithiothreitol (DTT), and 1% pharmalyte]. The suspension was centrifuged at 21000 *g* for 20 min and the supernatant was collected for further protein quantification. Protein content was determined using the method of Bradford (1976). A 10 μL aliquot of protein extract was mixed with 0.5 mL of a commercial color reagent (Bio-Rad Laboratories, Hercules, CA, United States) by a bovine serum albumin (BSA) standard. The absorbance was measured spectrophotometrically at 595 nm between 5 and 30 min after reaction.

### Two-Dimensional PAGE and Protein Analysis

An IPGPhor apparatus (GE Healthcare, Waukesha, WI, United States) was used for the first isoelectric focusing (IEF) described by Xu and Huang (2008). The extracts containing 300 μg of sample protein were used for IEF in immobilized pH gradient (IPG) strips (pH 3.0–10.0, linear gradient, 13 cm), formed by rehydrating strips for 12 h at room temperature in 250 μL of rehydration buffer (8 M urea, 2 M thiourea, 2% CHAPS, 1% DTT, 1% v/v IPG buffer, and 0.002% bromophenol blue). Following IEF, the IPG strips were equilibrated for 15 min twice at room temperature in equilibration buffer (50 mM Tris–HCl pH 8.8, 6 M urea, 30% glycerol, 2% SDS, and 1% DTT), then transferred to the same equilibration buffer containing 2.5% iodoacetamide instead of 1% DTT. The second dimension electrophoresis was run on a 12.5% SDS–polyacrylamide gel with a Hoefer SE 600 Ruby electrophoresis apparatus (GE Healthcare, Waukesha, WI, United States). The running conditions were 5 mA per strip for 30 min followed by 20 mA per strip for about 5 h. Gels were stained with Coomassie brilliant blue G-250 and scanned using a Personal Densitometer SI (63-0016-46, GE Healthcare, Waukesha, WI, United States).

Gel images analysis was performed by Progenesis software (Nonlinear Dynamics, Durham, NC, United States). Automatic default spot analysis settings were coupled with manual correction and editing of spot features. The spot volumes were normalized as a percentage of the total volume of all spots on the gel to correct the variability due to staining. Variance analysis of data was used to test the treatment effects on each transgenic line.

Selected protein spots were manually excised from gels and subjected to a trypsin digestion. The peptides were identified by MALDI-TOF-MS as described by Xu and Huang (2008). Data were searched against the National Center for Biotechnology Information (NCBI) database. Proteins containing at least two peptides with a confidence interval value >95% were considered to be successfully identified (Ma et al., 2016).

Protein functional classification was performed by Mapman software (Thimm et al., 2004) in combination with the criteria proposed by Bevan et al. (1998). The identified proteins were distributed to different subcellular location by SUBA (Tanz et al., 2013). Gene ontology (GO) for biological process, molecular function and cellular component was conducted by the agrigo database^1^; threshold was -log10 > 4 (Ma et al., 2016).

### Statistical Analysis

Data were analyzed using statistics software (SPSS 13.0; SPSS Inc., Chicago, IL, United States). Analysis of variance (ANOVA) was used to determine differences among treatment effects at a given treatment time. The means ± SE were calculated for each parameter. When a particular *F-*test was significant, means were tested with least significant difference (LSD) at a confidence level of 0.05.

## Results

### Physiological Effects of Elevated CO_2_

**FIGURE 1:**
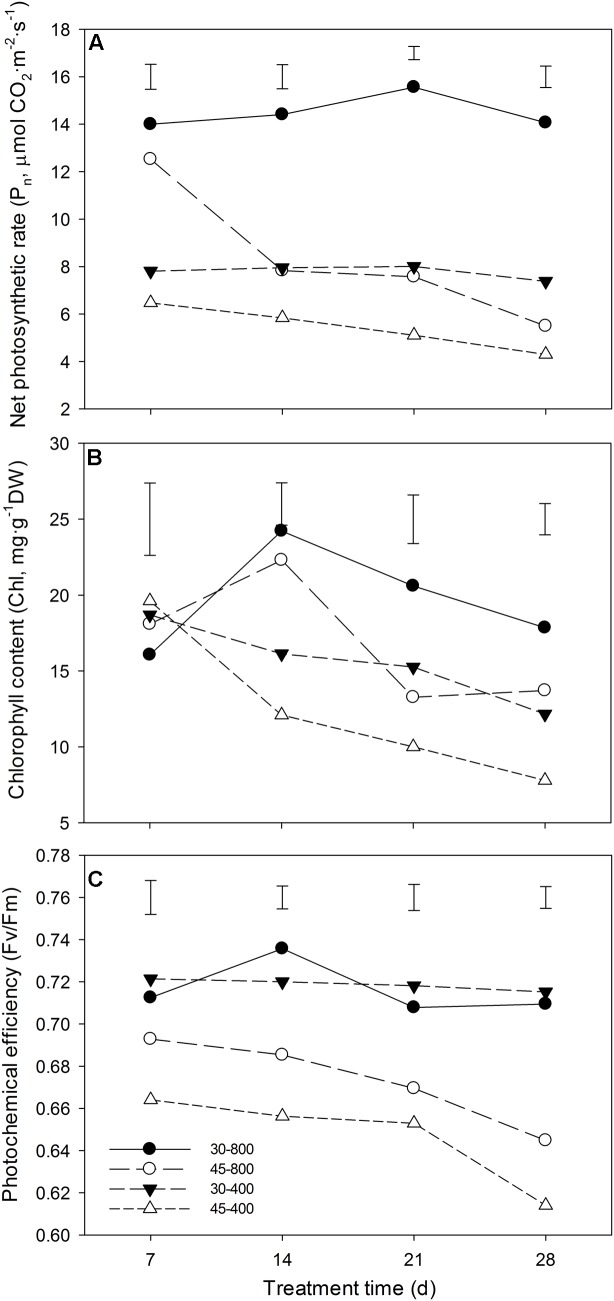
Effects of elevated CO_2_ concentration (800 μmol⋅mol^-1^ vs. 400 μmol⋅mol^-1^) on net photosynthetic rate (*P*_n_) **(A)**, chlorophyll content (Chl) **(B)** and **(C)** photochemical efficiency (*F*_v_/*F*_m_) in response to heat stress in bermudagrass. The treatments symbols are 30 and 45 for normal temperature control and heat stress and 400 and 800 for ambient CO_2_ and elevated CO_2_ concentrations, respectively. Vertical bars indicate significant difference based on LSD values (*P* ≤ 0.05) for the comparison among treatments.

Under normal temperature, elevated CO_2_ significantly increased *P*_n_ and Chl (**Figures 1A,B**) while it had no significant effects on *F*v/*F*m (**Figure 1C**). Under heat stress, plants grown at elevated CO_2_ had significantly higher *P*_n_ (**Figure 1A**), Chl (**Figure 1B**), and *F*_v_/*F*_m_ (**Figure 1C**) than that at ambient CO_2_ concentration.

### Effects of Elevated CO_2_ on Metabolic Profiles

**FIGURE 2:**
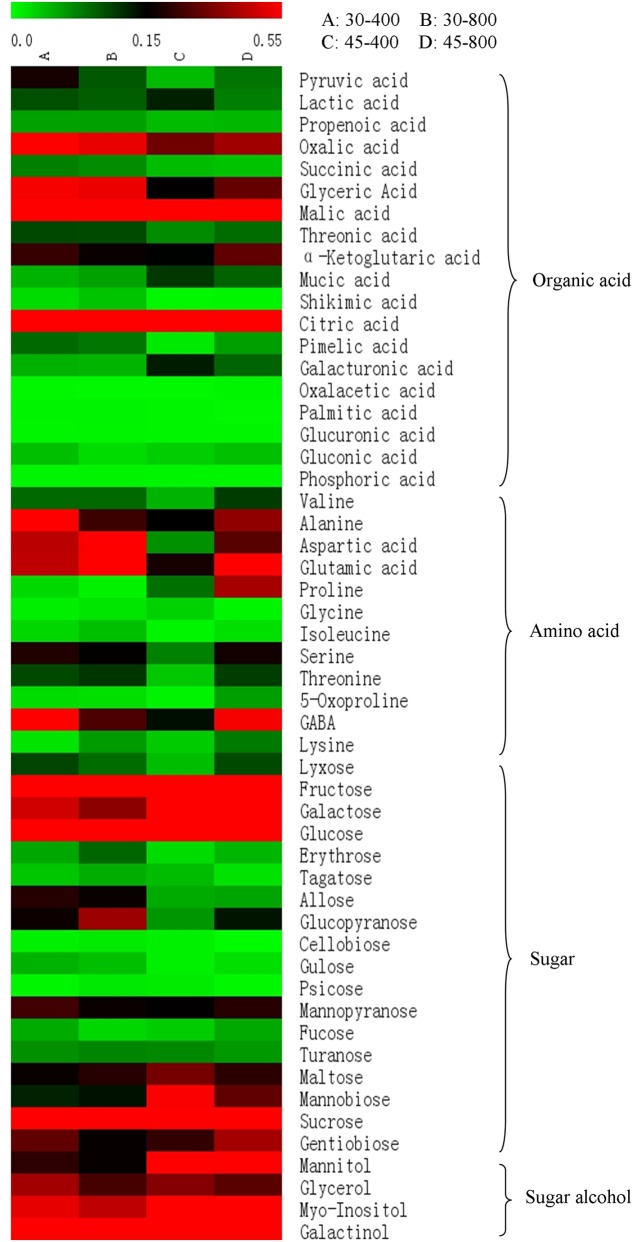
Heat map analysis of total 53 differentially expressed metabolites in response to different temperatures and CO_2_ concentrations. The treatments symbols are 30 and 45 for normal temperature control and heat stress and 400 and 800 for ambient CO_2_ and elevated CO_2_ concentrations, respectively.

A total of 53 metabolites, including 18 organic acids and phosphoric acid, 12 amino acids, 18 sugars and 4 sugar alcohols, in responsive to elevated CO_2_ and heat stress were identified and quantified by GC-MS. The name, RT, derivative and mass to charge (m/z) as well as the relative expression of each metabolite was presented in **Figure 2** and **Table 1**.

**Table 1:** Metabolites identified by GC-MS in response to different CO_2_ concentrations and temperatures in leaves of bermudagrass at 28 days of treatments.

Compound	RT	Derivative	m/z	Compound	RT	Derivative	m/z
Pyruvic acid	8.8	O-TMS^a^,MEOX1^b^	174	Erythrose	30.306	O-3TMS,MEOX1	205
Lactic acid	9.091	O-2TMS	147	Tagatose	31.15	O-5TMS,MEOX1	103
Propenoic acid	9.819	O-2TMS	147	Pimelic acid	32.978	O-3TMS	300
Alanine	10.24	N,O-TMS	116	Myo-Inositol	33.289	O-6TMS	305
Oxalic acid	11.267	O-2TMS	147	Allose	33.641	O-5TMS,MEOX1	319
Valine	13.331	N,O-TMS	144	Glucopyranose	33.802	O-6TMS	389
Glycerol	14.977	O-2TMS	205	Cellobiose	35.218	O-8TMS	204
Isoleucine	15.47	O-2TMS	117	Gulose	35.932	O-5TMS	204
Proline	15.547	N,O-TMS	142	Maltose	40.354	O-8TMS	204
Glycine	15.785	N,N,O-TMS	174	Galacturonic acid	40.611	O-5TMS	204
Succinic acid	16.059	O-2TMS	147	Mannobiose	40.972	O-8TMS	204
Glyceric Acid	16.4167	O-3TMS	147	Sucrose	42.414	O-8TMS	361
Serine	17.274	N,O,O-TMS	204	Galactinol	47.19	O-9TMS	204
Threonine	17.929	N,O,O-TMS	218	Gentiobiose	49.095	O-8TMS	204
Malic acid	20.565	O-3TMS	147	Psicose	26.779	O-5TMS,MEOX1	103
5-Oxoproline	21.276	O-2TMS	156	Mannopyranose	36.968	O-4TMS	204
Aspartic acid	21.328	O-3TMS	232	Fucose	37.747	O-4TMS	204
GABA	21.528	N,N,O-TMS	174	Glucuronic acid	31.323	O-3TMS	317
Lysine	21.611	N,N,O-TMS	174	Turanose	31.491	O-8TMS	361
Threonic acid	22.286	O-4TMS	292	Gluconic acid	31.59	O-6TMS	333
α-Ketoglutaric acid	22.666	O-2TMS,MEOX1	198	Palmitic acid	32.566	O-TMS	313
Glutamic acid	23.699	N,O,O-TMS	246	Oxaloacetic acid	32.701	O-3TMS	147
Lyxose	24.649	O-4TMS	103	Phosphoric acid	35.269	O-5TMS	357
Mucic acid	27.687	O-6TMS	333				
Shikimic acid	27.912	O-4TMS	204				
Citric acid	28.12	O-4TMS	273				
Fructose	29.109	O-5TMS,MEOX1	307				
Galactose	29.47	O-5TMS,MEOX1	319				
Glucose	29.611	O-5TMS,MEOX1	319				
Mannitol	30.236	O-6TMS	319				


*RT, retention time; m/z, mass to charge ratio; ^a^Trimethylsilyl derivative(s), ^b^Methoxime derivative(s).*

**FIGURE 3:**
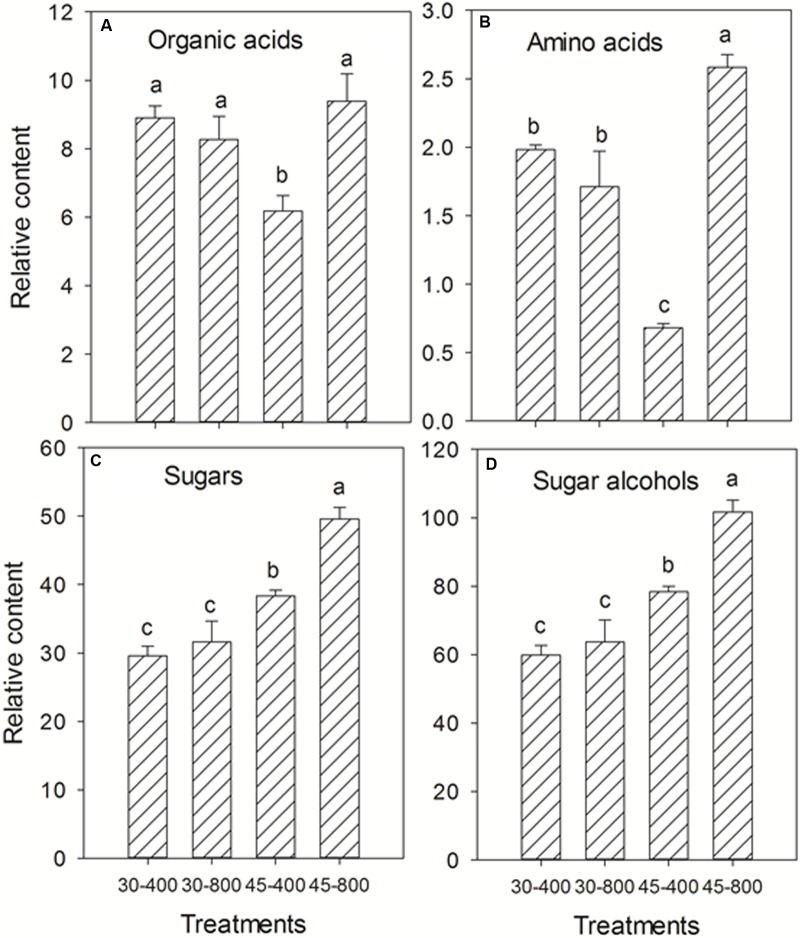
Effects of elevated CO_2_ concentration on total content of organic acids **(A)**, amino acids **(B)**, sugars **(C)**, and sugar alcohols **(D)** in response to heat stress in bermudagrass. The treatments symbols are 30 and 45 for normal temperature control and heat stress and 400 and 800 for ambient CO_2_ and elevated CO_2_ concentrations, respectively.

Total content of organic acids, amino acids, sugars, and sugar alcohols were presented in **Figure 3**. Under normal temperature, no effects of elevated CO_2_ were detected on total content of organic acids, amino acids, sugars and sugar alcohols compared with ambient CO_2_. Under heat stress, elevated CO_2_ resulted in significant increases in the content of organic acids, amino acids, sugars, and sugar alcohols by 52%, 2.79-fold, 29% and 30%, respectively (**Figure 3**).

**FIGURE 4:**
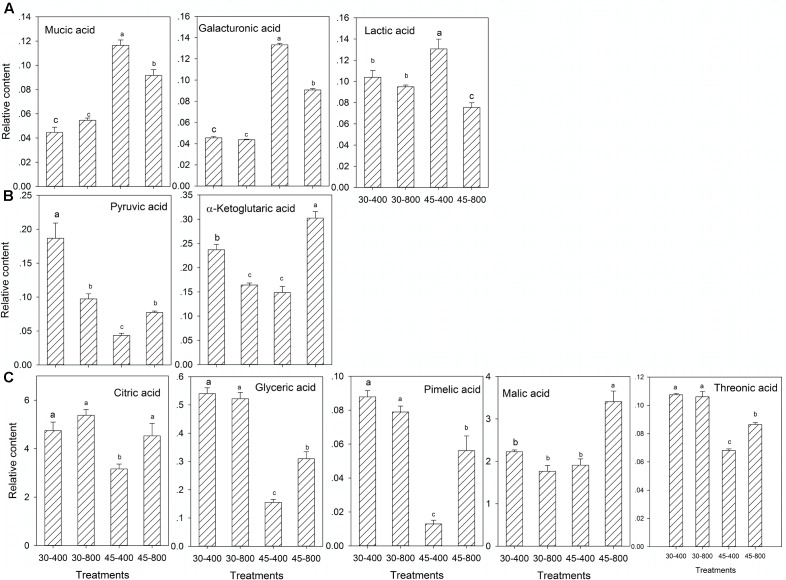
Effects of elevated CO_2_ concentration on organic acids in response to heat stress in bermudagrass. The treatments symbols are 30 and 45 for normal temperature control and heat stress and 400 and 800 for ambient CO_2_ and elevated CO_2_ concentrations, respectively. **(A)** No changes under 30–800 and down-regulation under 45–800; **(B)** No changes under 30–800 and up-regulation under 45–800; **(C)** Down-regulation under 30–800 and up-regulation under 45–800. Columns marked with different letters presented the significant differences based on LSD values (*P* ≤ 0.05) among treatments.

For organic acids, under heat stress, plants grown at elevated CO_2_ exhibited significantly lower content of mucic acid, galacturonic acid, lactic acid, but higher content of pyruvic acid, α-ketoglutaric acid, citric acid, glyceric acid, pimelic acid, malic acid and threonic acid compared to those with ambient CO_2_ (**Figures 4A–C**). Plants exposed to elevated CO_2_ had significantly lower content of pyruvic acid and α-ketoglutaric acid than those with ambient CO_2_ under normal temperature (**Figure 4B**).

**FIGURE 5:**
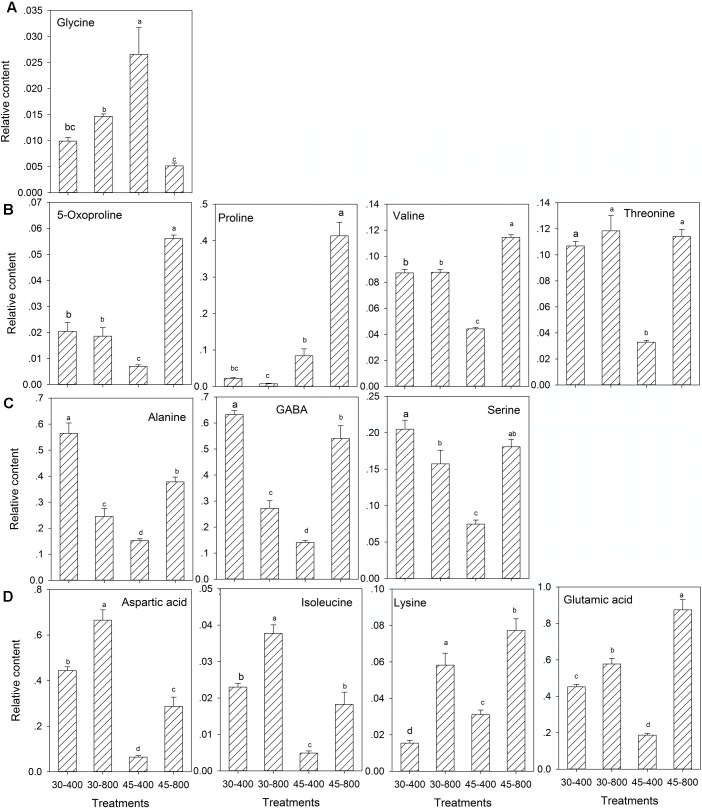
Effects of elevated CO_2_ concentration on amino acids in response to heat stress in bermudagrass. The treatments symbols are 30 and 45 for normal temperature control and heat stress and 400 and 800 for ambient CO_2_ and elevated CO_2_ concentrations, respectively. **(A)** No changes under 30–800 and down-regulation under 45–800; **(B)** No changes under 30–800 and up-regulation under 45–800; **(C)** Down-regulation under 30–800 and up-regulation under 45–800; **(D)** Up-regulation under both 30–800 and 45–800. Columns marked with different letters presented the significant differences based on LSD values (*P* ≤ 0.05) among treatments.

For amino acids, under heat stress, the content of all amino acids were increased by elevated CO_2_ except glycine compared with ambient CO_2_ (**Figure 5**). The content of alanine, GABA, and serine (**Figure 5C**) was significantly lower and the content of aspartic acid, isoleucine, lysine and glutamic acid (**Figure 5D**) was significantly higher in plants exposed to elevated CO_2_ compared with ambient CO_2_ treatments under normal temperature.

**FIGURE 6:**
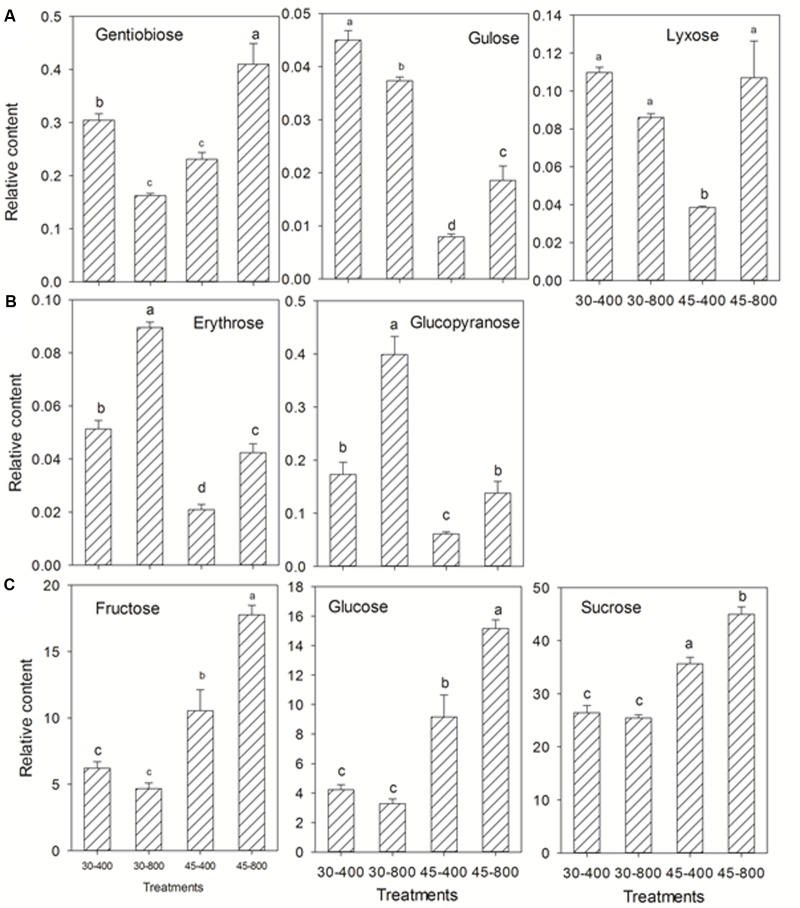
Effects of elevated CO_2_ concentration on sugars in response to heat stress in bermudagrass. The treatments symbols are 30 and 45 for normal temperature control and heat stress and 400 and 800 for ambient CO_2_ and elevated CO_2_ concentrations, respectively. **(A)** Down-regulation or no changes under 30–800 and down-regulation under 45–800; **(B)** Up-regulation under both 30–800 and 45–800; **(C)** No changes under 30–800 and up-regulation under 45–800. Columns marked with different letters presented the significant differences based on LSD values (*P* ≤ 0.05) among treatments.

**FIGURE 7:**
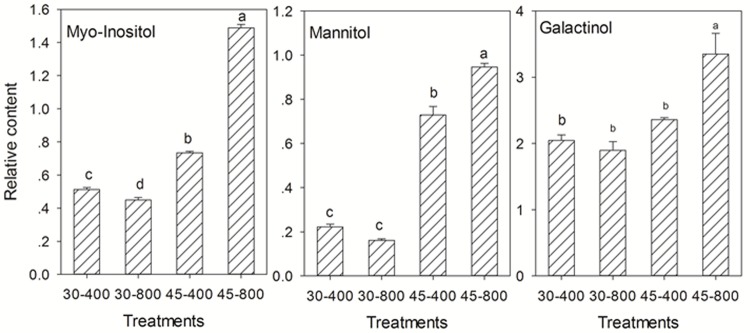
Effects of elevated CO_2_ concentration on sugar alcohols in response to heat stress in bermudagrass. The treatments symbols are 30 and 45 for normal temperature control and heat stress and 400 and 800 for ambient CO_2_ and elevated CO_2_ concentrations, respectively. Columns marked with different letters presented the significant differences based on LSD values (*P* ≤ 0.05) among treatments.

For sugars and sugar alcohols, the content of gentiobiose, gulose, lyxose (**Figure 6A**), and myo-inositol (**Figure 7**) was decreased while that of erythrose and glucopyranose (**Figure 6B**) was increased by elevated CO_2_ compared to plants grown at ambient CO_2_ concentration under normal temperature. Under heat stress, 8 out of 18 sugars, and two sugar alcohols (mannitol and galactinol) exhibited increases in the content in plants exposed to elevated CO_2_ compared with ambient CO_2_ (**Figures 6**, **7**).

**FIGURE 8:**
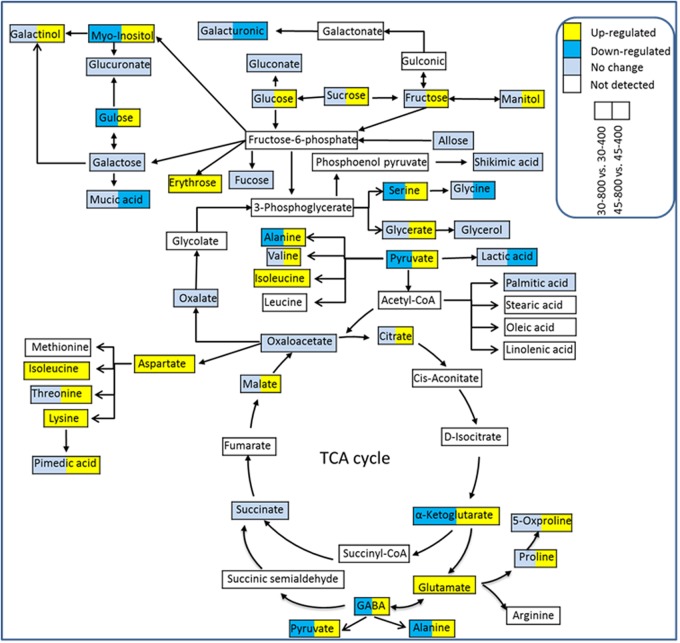
The metabolic pathways associated with differentially expressed metabolites. The treatments symbols are 30 and 45 for normal temperature control and heat stress and 400 and 800 for ambient CO_2_ and elevated CO_2_ concentrations, respectively.

Out of 53 identified metabolites, 43 were placed into the metabolic pathways associated with GABA shunt, TCA cycle, sugar and amino acid metabolism (**Figure 8**). These 43 metabolites included 16 organic acids, 12 amino acids, 11 sugars and 4 sugar alcohols. Under heat stressed conditions, elevated CO_2_ enhanced the accumulation of metabolites associated with GABA shunt, sugar and amino metabolisms.

### Proteomic Responses to Elevated CO_2_

**FIGURE 9:**
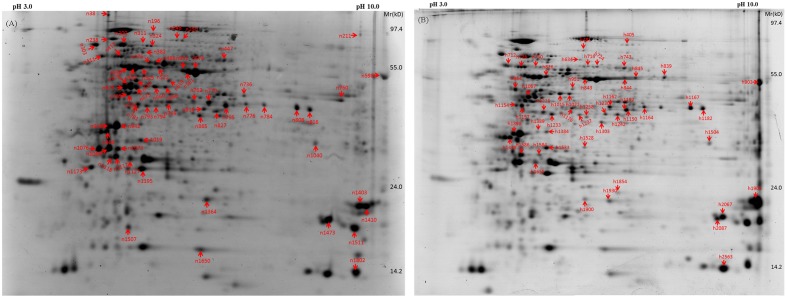
Representative gels of 2-D with differentially expressed proteins identified in bermudagrass grown under normal temperature **(A)** and heat stress **(B)** at 28 days of treatments. Labels of spots in each gel were consistent with **Table 2**.

A total of 70 and 53 protein spots were differentially expressed in leaves of bermudagrass due to elevated CO_2_ compared to those at ambient CO_2_ under normal and high temperature, respectively (**Figure 9** and **Table 2**).

**Table 2:** Differentially expressed proteins in response to different CO_2_ concentrations and temperatures by comparison between elevated CO_2_ and ambient CO_2_ in leaves of bermudagrass at 28 days of treatments.

Spot no.	Unipro. ID	Pro. name [species]	pI	MW	PM
n38	A0A0A9D510	Uncharacterized protein [*Arundo donax*]	11.472786	20592.018	2
n196	C0PFV4	Cytokinin inducible protease1 [*Zea mays*]	6.23571	102041	4
n211	A0A0A9R3Q6	Uncharacterized protein [*Arundo donax*]	9.6397934	10972.589	2
n238	C1K9J1	Heat shock protein 90 [*Zea mays*]	5.004387	80090	5
n248	Q8W0Q7	Methionine synthase protein [*Sorghum bicolor*]	5.930443	83788.72	6
n250	Q8W0Q7	Methionine synthase protein [*Sorghum bicolor*]	5.930443	83788.72	6
n300	X4Z319	Heat shock protein 70 [*Saccharum hybrid cultivar*]	5.127754	71034.47	7
n303	A4ZYQ0	Chloroplast heat shock protein 70 [*Pennisetum americanum*]	5.233284	73010.5	5
n311	K4AEH8	Glutathione S-transferase [*Arabidopsis thaliana*]	5.5051193	25757.487	2
n324	Q7SIC9	Transketolase, chloroplastic [*Zea mays*]	5.466347	72993.41	2
n382	K3XFX0	Phosphoglycerate mutase [*Arabidopsis thaliana*]	5.7386093	63645.472	4
n411	A0A096PMM2	Chaperonin-60 alpha [*Arabidopsis thaliana*]	5.0534134	63186.397	2
n417	C0PHP3	Putative TCP-1/cpn60 chaperonin family protein [*Zea mays*]	4.750603	44074.17	4
n447	A0A059PYZ3	Catalase [*Saccharum hybrid cultivar*]	6.5794296	56439.851	2
n476	A0A059Q9W7	ATP synthase subunit alpha, chloroplastic [*Neyraudia reynaudiana*]	5.7230148	55674.804	7
n486	A0A024GW45	ATP synthase subunit alpha, chloroplastic [*Lecomtella madagascariensis*]	5.865181	55704.87	6
n522	K3Z2G6	ATP synthase subunit alpha [*Setaria italica*]	5.7027206	55314.391	3
n525	A0A024GW49	ATP synthase subunit beta, chloroplastic [*Lecomtella madagascariensis*]	5.306984	53954.82	6
n530	A0A059Q9X1	ATP synthase subunit beta, chloroplastic [*Neyraudia reynaudiana*]	5.3016434	53997.843	8
n537	A0A024BLC0	ATP synthase subunit beta [*Pennisetum americanum*]	5.3069839	53910.765	7
n551	A0A0G2UKF5	Ribulose-1,5-bisphosphate carboxylase/oxygenase large subunit [*Orinus thoroldii*]	6.2336807	51506.568	6
n561	A0A059Q9V4	Ribulose-1,5-bisphosphate carboxylase/oxygenas [*Neyraudia reynaudiana*]	6.0360794	52724.927	6
n574	A0A0U5GUY4	Ribulose-1,5-bisphosphate carboxylase/oxygenase large subunit [*Neostapfiella perrieri*]	6.3398514	49871.655	7
n598	A0A059Q008	Elongation factor 1-alpha [*Saccharum hybrid cultivar*]	9.1394882	49276.993	3
n616	C0P699	Elongation factor Tu [*Zea mays*]	6.1954422	50776.346	3
n619	A0A077JG84	S-adenosylmethionine synthase [*Andropogon virginicus*]	5.5640793	43045.785	4
n660	K4AA01	Glyceraldehyde-3-phosphate dehydrogenase [*Setaria italica*]	6.1007004	46993.46	3
n666	K4AA01	Glyceraldehyde-3-phosphate dehydrogenase [*Setaria italica*]	6.1007004	46993.46	2
n673	K3Z5U9	Phosphoglycerate kinase [*Setaria italica*]	6.0720749	49686.217	4
n675	K3Z5U9	Phosphoglycerate kinase [*Setaria italica*]	6.072075	49686.22	3
n678	K3XH82	Phosphoglycerate kinase [*Setaria italica*]	8.4882584	50239.992	4
n705	B6T2L2	Sedoheptulose-1,7-bisphosphatase [*Zea mays*]	6.074532	41816.7	3
n706	K3YTN2	Glutamine synthetase [*Setaria italica*]	5.5121689	39158.048	2
n736	K3XHJ0	Aspartate aminotransferase [*Setaria italica*]	8.8027115	50210.455	4
n760	K3XHJ0	Aspartate aminotransferase [*Setaria italica*]	8.8027115	50210.455	4
n762	A0A096TAE3	Glyceraldehyde-3-phosphate dehydrogenase [*Zea mays*]	7.0012283	42856.79	2
n769	P0C1M0	ATP synthase subunit gamma, chloroplastic [*Zea mays*]	8.4372025	39789.807	5
n770	A0A096TAE3	Glyceraldehyde-3-phosphate dehydrogenase [*Zea mays*]	7.0012283	42856.79	2
n776	K3YS38	Glyceraldehyde-3-phosphate dehydrogenase [*Setaria italica*]	9.3867569	51746.214	2
n781	K3ZIS7	Fructose-bisphosphate aldolase [*Setaria italica*]	6.2657242	42104.979	5
n784	C_4_JBS8	Glyceraldehyde-3-phosphate dehydrogenase [*Zea mays*]	6.459267	36494.67	3
n793	C0PD30	Fructose-bisphosphate aldolase [*Zea mays*]	6.3739243	38146.552	4
n794	K3ZIS7	Fructose-bisphosphate aldolase [*Setaria italica*]	6.2657242	42104.979	5
n795	A0A096TAE3	Glyceraldehyde-3-phosphate dehydrogenase [*Zea mays*]	7.0012283	42856.79	2
n808	K3YIG5	Glyceraldehyde-3-phosphate dehydrogenase [*Setaria italica*]	6.9726028	36597.831	3
n814	A0A140GYJ8	Cysteine synthase C1 [*Arabidopsis thaliana*]	7.7287216	40549.937	2
n816	K3YIG5	Glyceraldehyde-3-phosphate dehydrogenase [*Setaria italica*]	6.9726028	36597.831	3
n827	K3Z7Q4	Malate dehydrogenase [*Setaria italica*]	8.229454	35523.89	3
n865	B6TEW2	Ferredoxin–NADP reductase, leaf isozyme [*Zea mays*]	8.372902	37506.14	2
n941	B4F9R9	Oxygen-evolving enhancer protein 1 [*Zea mays*]	5.539513	35079.65	5
n942	B4F9R9	Oxygen-evolving enhancer protein 1 [*Zea mays*]	5.539513	35079.65	5
n968	B6SQQ0	Inorganic pyrophosphatase [*Zea mays*]	5.788383	31736.97	2
n1019	J9QDZ6	Ascorbate peroxidase [*Saccharum* hybrid cultivar]	5.176033	27159.67	3
n1040	B4FT85	Isochorismate synthase 1 [*Zea mays*]	7.855827	29470.34	2
n1066	B4FNR1	Chlorophyll a-b binding protein 2 [*Zea mays*]	5.140251	27815.74	3
n1073	B6UG30	Triosephosphate isomerase [*Zea mays*]	6.139687	32392.87	2
n1076	B4FNR1	Chlorophyll a-b binding protein 2 [*Zea mays*]	5.140251	27815.74	2
n1117	B6SS26	Adenylate kinase [*Zea mays*]	6.790276	31139.68	2
n1118	K3YIW7	Adenosine monophosphate kinase [*Arabidopsis thaliana*]	7.6875992	31556.069	2
n1127	B6SUC_4_	Chlorophyll a-b binding protein 8 [*Zea mays*]	8.940819	28984.34	2
n1173	C_4_J9M7	2-cys peroxiredoxin BAS1 [*Zea mays*]	5.807823	28272.36	3
n1195	B6SSN3	Chlorophyll a-b binding protein 6A [*Zea mays*]	6.214775	26309.18	3
n1364	B4F9N4	Cytochrome b6-f complex iron-sulfur subunit [*Zea mays*]	8.5790482	24054.508	3
n1403	A0A0A6Z9F5	Photosystem I reaction center subunit II [*Saccharum hybrid cultivar*]	9.913979	21844.1	4
n1410	A0A0A6Z9F5	Photosystem I reaction center subunit II [*Saccharum hybrid cultivar*]	9.913979	21844.1	5
n1473	B6SPC1	Photosystem I reaction center subunit IV A [*Zea mays*]	9.786659	14893.9	4
n1507	A0A024GWT9	ATP synthase epsilon chain, chloroplastic [*Lecomtella madagascariensis*]	5.027992	15245.6	2
n1511	B4G259	Photosystem II Subunit Q [*Arabidopsis thaliana*]	9.771919	23132.72	4
n1650	A0A0A9IAK2	Ribulose bisphosphate carboxylase small chain [*Arundo donax*]	6.306633	14832.18	2
n1802	O65101	Photosystem I reaction center subunit VI, chloroplastic [*Zea mays*]	10.09834	14929.3	2
h405	B5AMJ8	Alpha-1,4 glucan phosphorylase [*Zea mays*]	6.8560715	94452.824	2
h481	Q8W0Q7	Methionine synthase protein [*Sorghum bicolor*]	5.9304428	83788.725	6
h634	A0A096QX48	Succinate dehydrogenase [ubiquinone] flavoprotein subunit [*Zea mays*]	6.0423813	63934.221	2
h712	A0A096PMM2	CPN60A [*Arabidopsis thaliana*]	5.0534134	63186.397	2
h716	A0A096RAX3	Malic enzyme [*Zea mays*]	8.0004501	67809.036	2
h724	A0A096RAX3	Malic enzyme [*Zea mays*]	8.0004501	67809.036	4
h730	C0PHP3	Putative TCP-1/cpn60 chaperonin family protein [*Zea mays*]	4.7506027	44074.173	4
h736	C0PHP3	Putative TCP-1/cpn60 chaperonin family protein [*Zea mays*]	5.4693375	64030.34	5
h743	A0A059PYZ3	Catalase [*Saccharum* hybrid cultivar R570]	6.5794296	56439.851	2
h826	A0A024GW49	ATP synthase subunit beta, chloroplastic [*Lecomtella madagascariensis*]	5.3069839	53954.82	6
h839	C5Z2J6	Catalase [*Sorghum bicolor*]	6.6157455	56841.332	3
h843	A0A0G2UKF5	Ribulose-1,5-bisphosphate carboxylase/oxygenase large subunit [*Orinus thoroldii*]	6.2336807	51506.568	6
h844	C5Z2J6	Catalase [Sorghum bicolor]	6.6157455	56841.332	3
h845	A0A059Q0R4	NADP-dependent glyceraldehyde-3-phosphate dehydrogenase [*Saccharum* hybrid cultivar R570]	6.8003159	53254.56	2
h903	A0A059Q008	Elongation factor 1-alpha [*Saccharum* hybrid cultivar R570]	9.1394882	49276.993	3
h951	A0A077JG84	S-adenosylmethionine synthase [*Andropogon virginicus*]	5.5640793	43045.785	4
h964	K3ZIK0	Rubisco activase [*Arabidopsis thaliana*]	6.1982193	47531.255	4
h1015	K4AA01	Glyceraldehyde-3-phosphate dehydrogenase [*Setaria italica*]	6.1007004	46993.46	3
h1022	K4AA01	Glyceraldehyde-3-phosphate dehydrogenase [*Setaria italica*]	6.1007004	46993.46	2
h1067	K3XH82	Phosphoglycerate kinase [*Setaria italica*]	8.4882584	50239.99	2
h1150	A0A096TAE3	Glyceraldehyde-3-phosphate dehydrogenase [*Zea mays*]	7.0012283	42856.79	2
h1154	B6T2L2	Sedoheptulose-1,7-bisphosphatase [*Zea mays*]	6.0745316	41816.7	4
h1157	B6T2L2	Sedoheptulose-1,7-bisphosphatase [*Zea mays*]	6.0745316	41816.7	3
h1162	A0A096TAE3	Glyceraldehyde-3-phosphate dehydrogenase [*Zea mays*]	7.0012283	42856.79	2
h1164	K3YS38	Glyceraldehyde-3-phosphate dehydrogenase [*Setaria italica*]	9.3867569	51746.214	2
h1167	K3YIG5	Glyceraldehyde-3-phosphate dehydrogenase [*Setaria italica*]	6.9726028	36597.831	3
h1176	P0C1M0	ATP synthase subunit gamma, chloroplastic [*Zea mays*]	8.4372025	39789.807	5
h1182	K3YIG5	Glyceraldehyde-3-phosphate dehydrogenase [*Setaria italica*]	6.9726028	36597.831	3
h1192	A0A096TAE3	Glyceraldehyde-3-phosphate dehydrogenase [*Zea mays*]	7.0012283	42856.79	2
h1211	K3ZIS7	Fructose-bisphosphate aldolase [*Setaria italica*]	6.2657242	42104.979	2
h1230	A0A140GYJ8	Cysteine synthase C1 [*Arabidopsis thaliana*]	7.7287216	40549.937	2
h1233	C0PD30	Fructose-bisphosphate aldolase [*Zea mays*]	6.3739243	38146.552	4
h1237	K3Z7Q4	Malate dehydrogenase [*Setaria italica*]	8.229454	35523.89	3
h1242	K3Z7Q4	Malate dehydrogenase [*Setaria italica*]	8.229454	35523.89	3
h1258	K3XJN7	Malate dehydrogenase [*Setaria italica*]	7.6717911	35479.854	3
h1303	B6TEW2	Ferredoxin–NADP reductase, leaf isozyme [*Zea mays*]	8.3729019	37506.14	2
h1384	C0PK05	Lactoylglutathione lyase [*Zea mays*]	5.8257675	32344.8	3
h1389	C0PK05	Lactoylglutathione lyase [*Zea mays*]	5.8257675	32344.804	3
h1398	B4F9R9	Oxygen-evolving enhancer protein 1 [*Zea mays*]	5.5395126	35079.65	5
h1438	K3YUP7	Pyrophosphorylase 6 [*Arabidopsis thaliana*]	5.7826157	31748.944	2
h1504	B4FT85	Isochorismate synthase 1 [*Zea mays*]	7.8558273	29470.34	2
h1528	K3Y8C6	Ascorbate peroxidase 4 [*Arabidopsis thaliana*]	8.1654739	38125.019	3
h1533	B6TVL8	APx2-Cytosolic Ascorbate Peroxidase [*Zea mays*]	5.2829514	27201.75	3
h1584	B4FQW0	Stem-specific protein TSJT1 [*Zea mays*]	5.233284	24666.251	2
h1586	B6SS26	Adenylate kinase [*Zea mays*]	6.7902756	31139.68	4
h1655	B6SUC_4_	Chlorophyll a-b binding protein 8 [*Zea mays*]	8.9408188	28984.34	3
h1854	A0A0B4J349	Peptidyl-prolyl cis-trans isomerase[*Zea mays*]	9.4011765	26443.981	3
h1900	B4F9N4	Cytochrome b6-f complex iron-sulfur subunit [*Zea mays*]	8.5790482	24054.508	3
h1905	A0A0A6Z9F5	Photosystem I reaction center subunit II [*Saccharum hybrid cultivar*]	9.9139786	21844.1	4
h1930	B4F9N4	Cytochrome b6-f complex iron-sulfur subunit [*Zea mays*]	8.5790482	24054.508	3
h2067	B6SPC1	Photosystem I reaction center subunit IV A [*Zea mays*]	9.7866592	14893.9	3
h2087	B6ST36	Chloroplast oxygen-evolving complex/thylakoid lumenal 25.6kDa protein [*Zea mays*]	9.3444595	26165.12	2
h2563	B4FAC2	Photosystem I reaction center subunit N [*Zea mays*]	9.2116928	15485.72	3


*n, normal temperature (30°C); h, heat stress (45°C); pI, isoelectric point; MW (kDa), molecular weight; PW, the number of unique peptides matched.*

**FIGURE 10:**
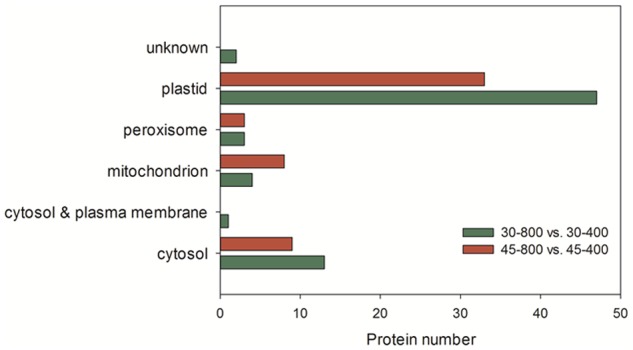
Subcellular location of identified proteins in response to different CO_2_ concentrations and temperatures. The treatments symbols are 30 and 45 for normal temperature control and heat stress and 400 and 800 for ambient CO_2_ and elevated CO_2_ concentrations, respectively.

**FIGURE 11:**
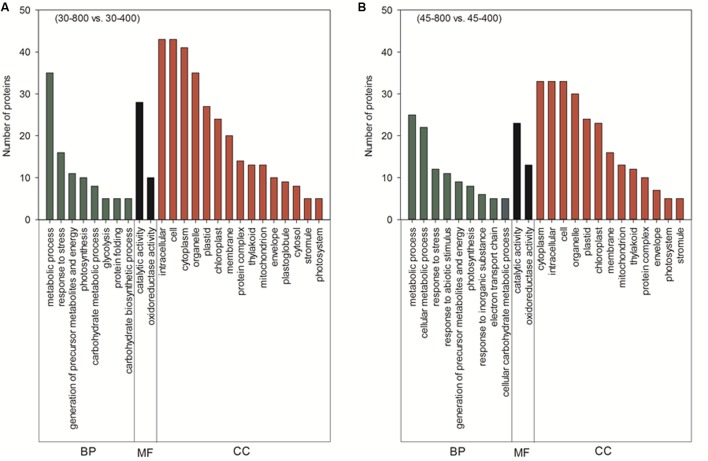
Cluster analysis from gene ontology (GO) analysis of differentially expressed proteins in response to different CO_2_ concentrations under normal temperature **(A)** and heat stress **(B)** in leaves of bermudagrass. The treatments symbols are 30 and 45 for normal temperature control and heat stress and 400 and 800 for ambient CO_2_ and elevated CO_2_ concentrations, respectively. BP, biological process; MF, molecular function; CC, cellular component.

Those 70 proteins up- or down-regulated by elevated CO_2_ under normal temperature were found in plastid (67.1%), cytosol (18.6%), mitochondrion (5.7%), peroxisome (4.3%), cytosol and plasma membrane (1.4%) and unknown locations (2.9%) (**Figure 10**). GO category enrichment showed that 70 proteins participated in various biological processes (metabolic process, response to stress, generation of precursor metabolites and energy, photosynthesis, carbohydrate metabolic process, glycolysis, protein folding, and carbohydrate biosynthetic process), molecular functions (catalytic activity and oxidoreductase activity) and cellular components (intracellular, cell, cytoplasm, organelle, plastid, chloroplast, membrane, protein complex, thylakoid, mitochondrion, envelope, plastoglobule, cytosol, stromule, and photosystem) (**Figure 11A**).

The majority of these 53 proteins up- or down-regulated by elevated CO_2_ under heat stress mainly distributed in plastid (62.3%) followed by cytosol (17%) (**Figure 10**). GO category enrichment indicated that the biological processes regulated by CO_2_ included cellular metabolic process, responses to stress, response to abiotic stimulus, generation of precursor metabolites and energy, photosynthesis, response to inorganic substance, electron transport chain, carbohydrate metabolism, molecular functions (catalytic activity and oxidoreductase activity) and cellular components (cytoplasm, intracellular, cell, organelle, plastid, chloroplast, membrane, mitochondrion, thylakoid, protein complex, envelope, photosystem, and stromule) (**Figure 11B**).

**FIGURE 12:**
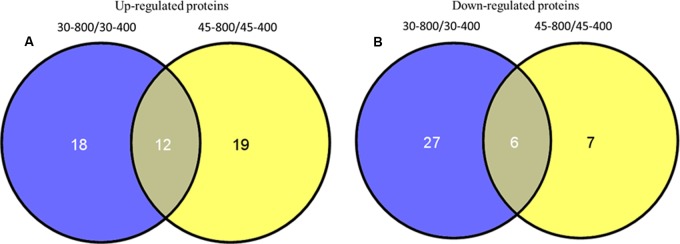
Venn analysis of up-regulated proteins **(A)** and down-regulated proteins **(B)** identified in bermudagrass at 28 days of treatments. The treatments symbols are 30 and 45 for normal temperature control and heat stress and 400 and 800 for ambient CO_2_ and elevated CO_2_ concentrations, respectively.

**FIGURE 13:**
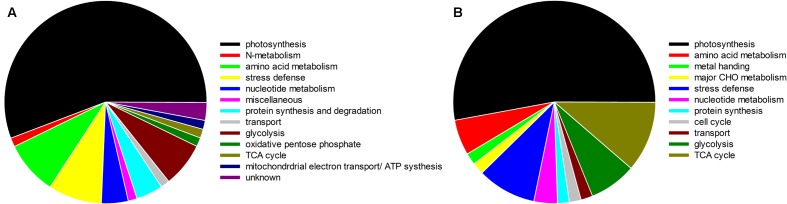
Functional classification of CO_2_ responsive proteins identified in bermudagrass grown under normal temperature **(A)** and heat stress **(B)** at 28 days of treatments.

Based on the Venn analysis, 18 and 19 differential proteins were up-regulated by elevated CO_2_ only under either normal temperature or heat stress, respectively (**Figure 12A**). Elevated CO_2_ caused 12 proteins to be up-regulated regardless of temperature (**Figure 12A**). A total of 40 proteins were down-regulated by elevated CO_2_ under normal and high temperature (**Figure 12B**). There were 27 proteins down-regulated under normal temperature and 7 proteins down-regulated under heat stress alone due to elevated CO_2_ treatment. The differentially expressed proteins in responses to elevated CO_2_ and heat stress were classified into different functional categories (**Figure 13**).

**FIGURE 14:**
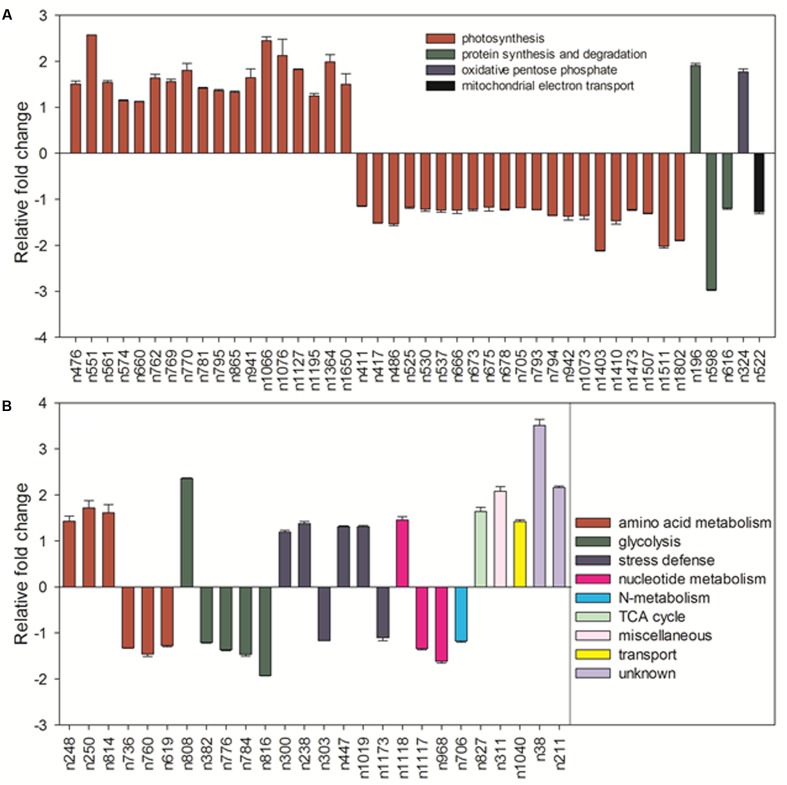
Comparison of protein abundance caused by elevated CO_2_ (800 μmol⋅mol^-1^) with ambient CO_2_ (400 μmol⋅mol^-1^) under normal temperature control (30°C). Charts are organized by the functional category of proteins involved in photosynthesis, protein synthesis and degradation, oxidative pentose phosphate and mitochondrial electron transport as shown in **(A)** as well as amino acid metabolism, glycolysis, stress defense, nucleotide metabolism, N-metabolism, TCA cycle, miscellaneous, transport and unknown proteins as shown in **(B)**. The values of the mean ± SE represent the relative expression fold change of proteins in response to elevated CO_2_ under normal temperature. Labels with ‘n’ in X-axle were same as **Table 2**.

Under normal temperature condition, the differential proteins caused by elevated CO_2_ compared with ambient CO_2_ were involved in photosynthesis (55.7%), followed by amino acid metabolism (8.6%), glycolysis (7.1%), protein synthesis and degradation (4.3%), stress defense (8.6%), nucleotide metabolism (4.3%), and the remaining (11.4%) including those unknown functions (**Figure 13A**). For proteins related to photosynthesis, significant increases in the relative fold change were found in ATP synthase subunit (ATPA, n476) by 1.5-fold, rubisco large subunit (RBCL, n551, n561, n574) by 1.1- to 2.6-fold, glyceraldehyde-3-phosphate dehydrogenase (GAPDH, n660, n762, n770, n795) by 1.1- to 1.4-fold, ATP synthase subunit gamma (ATPC, n769) by 1.6-fold, fructose-bisphosphate aldolase (FBA, n781) by 1.4-fold, ferredoxin-NADP reductase (FNR, n865) by 1.3-fold, oxygen-evolving enhancer protein (OEE, n941) by 1.6-fold, chlorophyll a-b binding protein (LHC, n1066, n1076, n1127, n1195) by 1.3- to 2.4-fold, cytochrome b6-f complex iron-sulfur subunit (PGR, n1364) by 2.0-fold, rubisco small chain (RBCS, n1650) by 1.5-fold (**Figure 14A**). Other 21 proteins involved in photosynthesis [(chaperonin-60 alpha, CPN60A), n411 by 1.14-fold; (cpn60 chaperonin family protein, CPN60B), n417 by 1.52-fold; n486, ATPA by 1.53-fold; n525, n530 and n537, ATPB by 1.17- to 1.23-fold; n666, GAPDH by 1.23-fold; (Phosphoglycerate kinase, PGK), n673, n675 and n678 by 1.17- to 1.21-fold; (Sedoheptulose-1,7-bisphosphatase, SBPase), n705 by 1.18-fold; n793 and n794, FBA by 1.22 – 1.35; n942, OEE by 1.37-fold; n1073, TIM by 1.36-fold; (Photosystem I reaction center subunit, Psa), PsaD, n1403 and n1410 by 1.47- to 2.11-fold; n1473, PsaE by 1.23-fold; n 1507, ATPE by 1.3-fold; n1511, PsbQ by 2.02-fold; n1802, PsaH by 1.89-fold] were significantly down-regulated by elevated CO_2_ under normal temperature compared with ambient CO_2_ (**Figure 14A**).

Among proteins associated with the function of protein synthesis and degradation, cytokinin inducible protease (CLPC, n196) had a 1.9-fold up-regulation and the other two [(elongation factor, EF), n598 and n616] with 1.2- to 3.0-fold down-regulation compared with ambient CO_2_ under normal temperature. Transketolase (n324) involved in oxidative pentose phosphate showed a 1.8-fold increase in response to elevated CO_2_ (**Figure 14A**). Proteins involved in amino acid metabolism exhibited increases in methionine synthase protein (MS, n248, n250) by 1.4- to 1.7-fold and cysteine synthase C1 (CSase, n814) by 1.6-fold as well as decreases in aspartate aminotransferase (ASP, n736, n760) by 1.3- to 1.5-fold and S-adenosylmethionine synthase (SAMS, n619) by 1.3-fold in plants grown at elevated CO_2_ compared with ambient CO_2_. GAPDH associated with glycolysis were all down-regulated by 1.4- to 1.9-fold (n382, n776, n784) under elevated CO_2_ except n808, which was increased by 2.4-fold under elevated CO_2_ concentration. There were six proteins associating with stress defense of which four proteins [Heat shock protein (Hsp) 90, n238; Hsp 70, n300; Catalase, n447; Ascorbate peroxidase, n1019] were up-regulated by 1.2- to 1.4-fold and tow proteins (Hsp 70, n303; 2-cys peroxiredoxin BAS1, n1173) were down-regulated by 1.1- to 1.2-fold under elevated CO_2_ concentration (**Figure 14B**).

**FIGURE 15:**
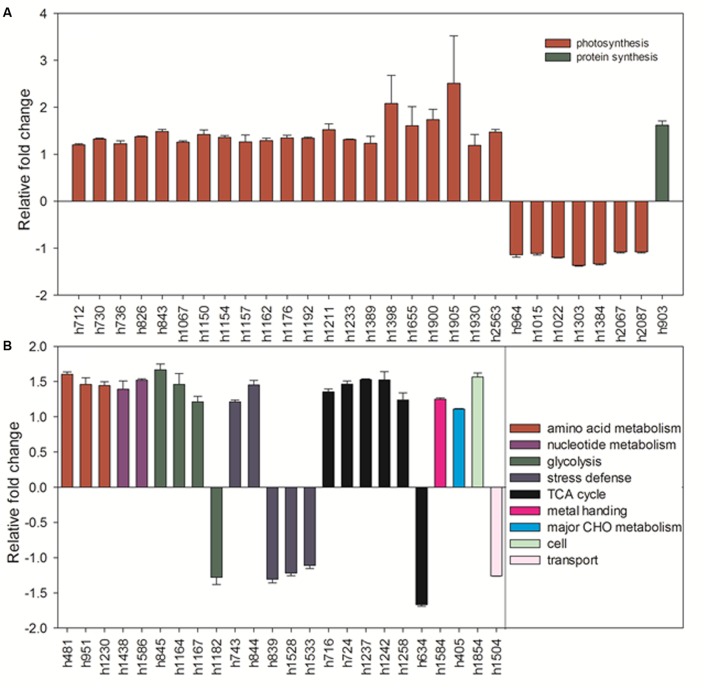
Comparison of protein abundance caused by elevated CO_2_ (800 μmol⋅mol^-1^) with ambient CO_2_ (400 μmol⋅mol^-1^) under heat stress (45°C). Charts are organized by the functional category of proteins involved in photosynthesis and protein synthesis as shown in **(A)** as well as amino acid metabolism, glycolysis, stress defense, TCA cycle, metal handing, major CHO metabolism, cell and transport as shown in **(B)**. The values of the mean ± SE represent the relative expression fold change of proteins in response to elevated CO_2_ under heat stress. Labels with ‘h’ in X-axle were same as **Table 2**.

Under heat stress, elevated CO_2_-regulated proteins were classified the into following functional categories: photosynthesis (52.8%), TCA cycle (11.3%), stress defense (9.4%), glycolysis (7.5%), amino acid metabolism (5.7%), nucleotide metabolism (3.8%), metal handling (1.9%), major CHO metabolism (1.9%), protein synthesis (1.9%), cell cycle (1.9%) and transport (1.9%) (**Figure 13B**). Proteins associated with photosynthesis were mainly up-regulated by elevated CO_2_ under heat stress, including CPN60A (h712) by 1.2-fold, CPN60B (h730, h736) by 1.2- to 1.3-fold, ATP synthase subunit beta (ATPB, n826) by 1.4-fold, RBCL (h843) by 1.5-fold, PGK, (h1067) by 1.3-fold, GAPDH (h1150, h1162, h1162, h1192) by 1.3-fold, SBPase (h1154, h1157) by 1.3-fold, ATPC (h1176) by 1.3-fold, FBA (h1211, h1233) by 1.3- to 1.5-fold, OEE (h1398) by 2.1-fold, LHC (h1655) by 1.6-fold, PGR (h1900, h1930) by 1.2- to 1.7-fold, PsaD (h1905) by 2.5-fold, photosystem I reaction center subunit N (PsaN, h2563) by 1.5-fold (**Figure 15A**). One protein associated with protein synthesis was upregulated by elevated CO_2_ under heat stress (**Figure 15A**). All proteins involved in amino acid metabolism and nucleotide metabolism were up-regulated by elevated CO_2_ under heat stress, including MS (1.6-fold), SAMS (1.5-fold), CSase (1.4-fold), pyrophosphorylase 6 (PPa6, 1.7-fold), adenylate kinase (ADK, 1.5-fold). Most TCA cycle related proteins [(Malic enzyme, ME), h716 and h724] by 1.4- to 1.5-fold; [(Malate dehydrogenase, MDH), h1237, h1242 and h1258] by 1.2- to 1.5-fold)] were up-regulated by elevated CO_2_ compared with ambient CO_2_ during heat stress (**Figure 15B**).

## Discussion

Previous studies have shown positive effects of elevated CO_2_ on plant growth of C_4_ species under optimal temperature conditions (Huang and Xu, 2015). In this study, elevated CO_2_ significantly improved physiological activities of C_4_ bermudagrass under heat stress or mitigated heat stress damages, as manifested by physiological indexes, including higher leaf *P*_n_, *F*_v_/*F*_m_ and Chl. The positive physiological effects were associated with changes in various metabolic pathways regulated by elevated CO_2_. Metabolic and proteomic analysis in this study indicated that the underlying mechanisms of elevated CO_2_-mitigation of heat stress were mainly related to photosynthesis, respiration (glycolysis and TCA cycle), amino acid metabolism, and GABA shunt, and some of the metabolic factors regulated by elevated CO_2_ in the C_4_ grass species, bermudagrass, in this study are in common and some are different from those previously found in C_3_ grass species (Yu et al., 2014; Burgess and Huang, 2016). Due to the large number of metabolites and the complexity of metabolic pathways involved in CO_2_ effects, the following sections focused on the discussion of unique or different metabolic pathways found in bermudagrass in our study from those findings previously reported in other C_3_ plant species.

### Proteins and Metabolites in Photosynthesis Regulated by Elevated CO_2_ under Heat Stress

**FIGURE 16:**
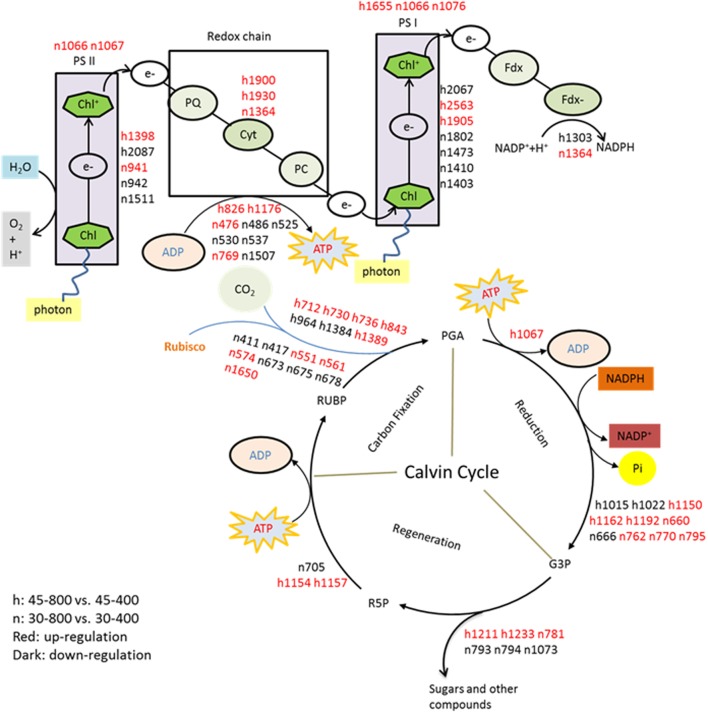
The metabolic pathways associated with differentially expressed proteins. The treatments symbols are 30 and 45 for normal temperature control and heat stress and 400 and 800 for ambient CO_2_ and elevated CO_2_ concentrations, respectively. Labels with ‘n’ or ‘h’ were same as **Table 2**. RuBP, Ribulose 1, 5-bisphosphate; R5P, Ribulose 5-phosphate; Rubisco, Ribulose 1, 5-bisphosphate carboxylase/oxygenase; PGA, 3-phosphoglyceric acid; G3P, Glyceraldehyde 3-phosphate; PC, Plastocyanin; PQ, Plastoquinone; Fd, Ferredoxin; Cyt, Cytochrome complex.

In our present study, 67 out of 123 proteins (54.5%) associated with photosynthetic pathways, including proteins involved in electron transport chain and Calvin cycle, were responsive to elevated CO_2_ concentration under normal and high temperatures, as shown by the decrease or increase in their abundance (**Figure 16**). Under heat stress, the majority of proteins involved in photosynthesis exhibited accumulation in response to elevated CO_2_ concentration, such as ATP synthase subunit (h826, h1176), photosystem I reaction center subunit [(PsaD, h1905) and (PsaN, h2563)] in light reactions of photosynthesis, and fructose-bisphosphate aldolase (FBA, h781, h1211, h1233), phosphoglycerate kinase (PGK, h1067) and sedoheptulose-1,7-bisphosphatase (SBPase, h1154, h1157) in Calvin cycle (**Figure 15**). FBA is a primary enzyme involved in the sixth reaction of Calvin cycle to convert fructose 1,6-bisphosphate into glyceraldehyde-3-phosphate (G3P) and dihydroxyacetone phosphate as well as ATP (Abbasi and Komatsu, 2004). FBA content at the level of protein significantly increased under elevated CO_2_ in C3 tall fescue under heat stress (Yu et al., 2014) and creeping bentgrass (*Agrostis stolonifera*) under both well-water and drought stress (Burgess and Huang, 2016). SBPase functions as a bisphosphatase enzyme catalyzing sedoheptulose 1,7-bisphosphate dephosphorylation to sedoheptulose-7-phosphate during the regeneration phase of Calvin cycle (Raines et al., 1999). Overexpression of SBPase in C_3_ tobacco (*Nicotiana tabacum*) had higher photosynthesis at elevated CO_2_ compared with that at ambient CO_2_ under field conditions (Rosenthal et al., 2011). The benefits of SBPase on the stimulation of photosynthesis depended on light intensity (Lefebvre et al., 2005; Rosenthal et al., 2011). Therefore, our study and another case in creeping bentgrass, conducted in light saturated growth chambers, the abundance of SBPase was enhanced by elevated CO_2_ under abiotic stresses (Burgess and Huang, 2016). FBA is a primary enzyme involved in the sixth reaction of Calvin cycle to convert fructose 1,6-bisphosphate into glyceraldehyde-3-phosphate (G3P), dihydroxyacetone phosphate and ATP (Abbasi and Komatsu, 2004). In addition, FBA could directly affect ribulose-1,5-bisphosphate (RuBP) regeneration which actions as substrate of carbon fixation (Taiz and Zeiger, 2010). FBA content at the level of protein showed the grater accumulation in elevated CO_2_ in C_3_ tall fescue under heat stress (Yu et al., 2014) and creeping bentgrass (*Agrostis stolonifera*) under both well-water and drought stress (Burgess and Huang, 2016). PGK is a major enzyme catalyzing the phosphorylation of 3-phosphoglycerate to produce 1, 3-bisphosphoglycerate and ADP which is one of vital steps regenerating RuBP during Calvin cycle (Bernstein et al., 1997). The regulation of FBA and PGK induced by elevated CO_2_ indicated that elevated CO_2_ availability in atmosphere could be helpful for sustaining ATP supply and RuBP regeneration for plant growth under heat stress. ATP synthase is a critical enzyme for creating energy storage molecule ATP. Under high CO_2_ availability, ATP synthase was found to decline in wheat grain (Högy et al., 2009). To our knowledge, our case is the first report on the abundance of ATP synthase and PGK in response to elevated CO_2_ were found in C_4_ plant species grown under heat stress. Our previous study in C_3_ plant species found differential responses of photosynthesis-related proteins to elevated CO_2_ different from found in bermudagrass in our study. In tall fescue, the abundance of ATP synthase subunit and PGK did not change in response to elevated CO_2_ under heat stress (Yu et al., 2014). The increase in abundance and activity of a single or some enzyme(s) during photosynthesis could enhance carbon assimilation (Rosenthal et al., 2011). Taken together, the enhanced accumulation of proteins involved in photosynthesis by elevated CO_2_ under heat stress in bermudagrass suggested that elevated CO_2_ could help to maintain photosynthesis to withstand the adverse environments as various proteins are involved in the light harvesting, electron transport, and carbohydrate assimilation processes of photosynthesis.

Other proteins related to photosynthesis such as GAPDH, OEE, PGR exhibited the enhanced expression in plants grown at elevated CO_2_ concentration under both temperatures in our study. GAPDH could convert G3P to D-glycerate 1,3-bisphosphate as well as mediating the formation of NADH and ATP (Tristan et al., 2011). It has multiple functions, such as two chloroplastic forms playing photosynthetic function locating in chloroplast and one cytosolic form participating in glycolysis in higher plants (Sparla et al., 2005; Tarze et al., 2007). In chloroplasts, GAPDH catalyzes a reaction of NADPH-consuming which is regulated by light utilizing thioredoxins and metabolites during Calvin cycle (Sparla et al., 2005). Various stresses caused the decline in chloroplastic GAPDH whereas stress-tolerant species exhibited higher GAPDH abundance than stress-sensitive plants, such as creeping bentgrass under heat stress (Xu and Huang, 2010a; Merewitz et al., 2011), salinity stress (Xu and Huang, 2010b) and drought stress (Burgess and Huang, 2016). Plants with lower GAPDH abundance were generally associated with decreased photosynthetic capacity resulted from reduced RuBP regeneration rate, followed with the decline in accumulation of photosynthetic products (Price et al., 1995; Burgess and Huang, 2016). However, elevated CO_2_ had no effects on chloroplastic GAPDH abundance under heat stressed condition but caused significant decrease under non-stressed control plants (Yu et al., 2014). Overall, our study suggested that enhanced abundance of photosynthesis-related proteins could contribute to the improved photosynthetic activities by elevated CO_2_, particularly under heat stress, which could be reflected with improved *P*_n_ and increased content of sugars, such as fructose, glucose, sucrose, erythrose, and glucopyranose.

### Proteins and Metabolites in Respiration Regulated by Elevated CO_2_ under Heat Stress

It has been widely known that glycolysis and TCA cycle are vital pathways for energy supply, amino acid synthesis and various other biological processes in plants (Fernie et al., 2004; Ma et al., 2016). As substrate of photosynthesis for carboxylation, plants grown at elevated CO_2_ tended to accumulate the larger amount of non-structural carbohydrates (Yu et al., 2012a; Song et al., 2014). Most monosaccharides (glucose, fructose, galactose, etc.) as substrate or intermediates play vital roles during glycolysis. Glycolysis pathway could convert glucose into pyruvate via a series of intermediate metabolites and cytosolic GAPDH is one of essential enzymes catalyzing the sixth step of respiratory glycolysis to convert G3P to 1, 3-bisphosphateglycerate (1, 3-BPG) which is one of the most important reactions during the glycolytic pathway (Mijeong et al., 2000). The increase of pyruvic acid (pyruvate) as the product of glycolysis, followed by the enhanced content of valine, isoleucine and alanine, was partly due to elevated CO_2_-caused accumulation of glucose under heat stress in our study, since those metabolites are all derived from glucose. In C_3_ tall fescue, we also observed the significant increases in valine and alanine but not for isoleucine resulted from elevated CO_2_ under heat stress (Yu et al., 2012a). During the pathway of glycolysis, the abundance of GAPDH in cytosol (n776, n784, n816 except n808) and phosphoglycerate mutase (PGAM, n382) exhibited the down-regulation in response to elevated CO_2_ rather than ambient CO_2_ under normal temperature, while under heat stressed conditions elevated CO_2_ caused up-regulation in GAPDH (h845, h1164, h1167 except h1182) in bermudagrass (**Figure 16**). GAPDH might serve as a provider of additional energy for plant growth and development under stressed conditions and stress tolerance could be enhanced by improved abundance of GAPDH to cope with environmental stresses (Mijeong et al., 2000; Bertrand et al., 2007). In C_3_ plants, no consistent changes were found due to variations in plant species. For example, in tall fescue and creeping bentgrass, the abundance of cytosolic GAPDH exhibited either no changes or decrease under elevated CO_2_ and heat stressed condition (Yu et al., 2014; Burgess and Huang, 2016). Kappachery et al. (2015) found that GAPDH gene-silenced lines showed more sensitive traits to drought stress than non-silenced lines in potato (*Solanum tuberosum*). By contrast, the higher shoot length and weight were detected in GAPDH overexpression transgenic plants compared with wild-type plants (Kappachery et al., 2015). In the level of transcription in potato, cytosolic GAPDH RNA accumulation was also increased under biological stress (Laxalt et al., 1996). Therefore, in our study, the higher abundance of GAPDH caused by elevated CO_2_ was beneficial for energy supply to support plant growth under heat stress.

Malate dehydrogenase (MDH) acts as an enzyme to catalyze the oxidation of malate to oxaloacetate via the reduction of NAD^+^ to NADH in mitochondrial matrix during TCA cycle (Musrati et al., 1998). Environmental stresses including drought (Burgess and Huang, 2016), heat (Xu and Huang, 2010a), salinity (Xu et al., 2010) and Al-stress (Ramírez-Benítez et al., 2008) have been shown to decrease the level of MDH in various plant species. However, limited studies about MDH were found in plants grown at elevated CO_2_ concentrations, especially under stressed conditions (Burgess and Huang, 2016). In this study, elevated CO_2_-responsive MDH (n827, h1237, h1242, h1258) involved in TCA cycle exhibited up-regulated expression regardless of temperatures, suggesting that CO_2_ inhibited the heat-induced reduction in MDH to catalyze the enhanced malate (malic acid) to oxaloacetate (oxaloacetic acid) during malate metabolism.

### Amino Acid Metabolism and GABA Shunt Regulated by Elevated CO_2_ under Heat Stress

In addition to function in TCA cycle, MDH also participates in the process of amino acid synthesis due to the relations among malate, oxaloacetate and aspartate (Musrati et al., 1998; Wen et al., 2015). Several amino acids including aspartate (aspartic acid), methionine, threonine, isoleucine, lysine derived from oxaloacetate and aspartate is the precursor of methionine, threonine, isoleucine and lysine (Muehlbauer et al., 1994). Along with the significant increase in malic acid and aspartic acid, the content of threonine, isoleucine and lysine were stimulated by elevated CO_2_ during heat stress. Furthermore, the content of alanine, valine and serine were also enhanced by elevated CO_2_ compared with ambient CO_2_ under heat stress. Alanine, valine and serine are used for synthesis of several proteins and associated with many metabolic processes (Bourguignon et al., 1999). The stimulation of elevated CO_2_ concentration on the content of alanine, valine and serine was found in other species under abiotic stresses, as previously reported in C_3_ grass species under heat stress (Yu et al., 2012a) and tree seedlings under drought stress (Tschaplinski et al., 1995). The increase in synthesis of both alanine and valine in present study is directly associated with the higher content of pyruvate (pyruvate acid) which is the final product of glycolysis (Schulzesiebert et al., 1984). Superior stress tolerance has been reported with the higher content of alanine, valine and serine as well as other amino acids such as GABA, glutamic acid, proline and 5-oxoproline involved in the GABA shunt pathway in plant species, including perennial grasses (Merewitz et al., 2012; Xu et al., 2013; Shi et al., 2014; Li et al., 2016a,b). The GABA shunt was considered to be a part of the TCA cycle during respiration besides its central role in primary carbon and nitrogen metabolism (Fait et al., 2008). In tall fescue, the content of GABA was significantly decreased by elevated CO_2_ under high temperature (Yu et al., 2014). While, in bermudagrass of this case, GABA and glutamic acid exhibited the opposite response to elevated CO_2_ under heat stress. Increased GABA caused the enhanced content of alanine and pyruvate which was turned into TCA cycle and proline metabolism. The content of all amino acids except arginine during GABA shunt was increased by elevated CO_2_ under heat stress suggesting a predominant role of elevated CO_2_ in carbon and nitrogen metabolism in C_4_ bermudagrass.

Proteins including methionine synthase (MS), cysteine synthase (CSase) and S-adenosylmethionine synthase (SAMS) associated with amino acid metabolism were up-regulated by 1.4- to 1.6-fold by elevated CO_2_ under heat stress. MS and SAMS serve as regulators in the synthesis and degradative pathways of various amino acids (Bohnert and Jensen, 1996). It was detected by the same proteomic analysis that many proteins involved in amino acid metabolism accumulated more or degraded less in stress-tolerant plants, such as MS and SAMS (Merewitz et al., 2011). CSase functions in the final strep in cysteine synthesis in plants. Plants with overexpressing CSase gene displayed high tolerance to toxic environmental pollutants, such as sulfur dioxide and sulfite (Noji et al., 2001), cadmium toxicity (Harada et al., 2001) in tobacco and aluminum toxicity in rice (Yang et al., 2007). The accumulation of many amino acids as well as proteins involved in amino acid metabolism in this study could contribute to elevated CO_2_-improved heat tolerance.

In summary, elevated CO_2_ concentration suppressed heat-induced damages in bermudagrass, as shown by the increased *P*_n_, Chl and *F*_v_/*F*_m_. The improvement of heat tolerance under elevated CO_2_ could be associated with some important metabolic pathways during which proteins and metabolites were up-regulated, including proteins, sugars and/or amino acids involved in light reaction (ATP synthase subunit and photosystem I reaction center subunit) and carbon fixation of photosynthesis (GAPDH, FBA, PGK, SBPase and sugars), glycolysis (GAPDH, glucose, fructose and galactose) and TCA cycle (pyruvic acid, malic acid and MDH) of respiration, amino acid metabolism (aspartic acid, methionine, threonine, isoleucine, lysine, valine, alanine and isoleucine) as well as the GABA shunt (GABA, glutamic acid, alanine, proline and 5-oxoproline). The molecular factors and mechanisms underlying the metabolic changes caused by elevated CO_2_ during plant responses to heat stress require further investigation.

## Author Contributions

JY and BH designed the experiments and wrote the manuscript. JY and RL conducted the experiments. NF helped with the sample analysis. ZY arranged the experiments and did the data analysis.

## Conflict of Interest Statement

The authors declare that the research was conducted in the absence of any commercial or financial relationships that could be construed as a potential conflict of interest.

## Footnotes

1 http://bioinfo.cau.edu.cn/agriGO/analysis.php
